# Effects of Forest Fire on Non-Structural Carbohydrates and Carbon, Nitrogen, and Phosphorus of *Pinus yunnanensis*

**DOI:** 10.3390/plants14233637

**Published:** 2025-11-28

**Authors:** Xiaoyong Fu, Weili Kou, Lili Wei, Jiangxia Ye, Qiuhua Wang

**Affiliations:** 1 College of Big Data and Intelligent Engineering, Southwest Forestry University, Kunming 650224, China; 2 Key Laboratory of National Forestry and Grassland Administration on Forestry and Ecological Big Data, Southwest Forestry University, Kunming 650224, China; 3 Yunnan Forestry Technological College, Kunming 650224, China; 4 College of Forestry, Southwest Forestry University, Kunming 650224, China; 5 Key Laboratory of Forest Disaster Warning and Control in Yunnan Province, Southwest Forestry University, Kunming 650224, China

**Keywords:** tree physiology, fire disturbance, nonstructural carbohydrate, stoichiometry, central Yunnan Province, *Pinus yunnanensis*

## Abstract

Fire is a crucial ecological factor, however, the organ-level effects of wildfire on plant non-structural carbohydrates (NSC) and stoichiometric characteristics are poorly understood. We studied the effects of fire on sugar, starch, carbon (C), nitrogen (N), and phosphorus (P) content in the needles, branches, stems, and fine roots of fire-damaged *Pinus yunnanensis* trees (F-trees). Starch, sugar, and NSC content were higher in the roots and needles of F-trees than in that of undamaged individuals (H-trees). C, N, and P content and stoichiometry characteristics of F-trees also differed significantly from H-trees. The N/P content of both F-tree and H-tree needles were <14 prior to forest fire disturbance and increased thereafter, indicating that the N limitation increased after fire disturbance. In addition, F-tree physiology did not return to control levels nine months after fire disturbance, indicating that more time is needed to recover. These findings suggest that forest management practices that increase soil N content will likely accelerate post-fire recovery. Because fire frequency and intensity will continue to increase in response to climate change, our results are of great value in improving our understanding of post-fire ecosystem dynamics.

## 1. Introduction

Fire is a significant disturbance to forest ecosystems, and has a great impact on the composition, structure, and function of forest vegetation. Between 1949 and 2015, 705,000 forest fires occurred across China, and affected 38.74 million hectares of forest [1]. Yunnan province is located on the southwest border of China, with complex terrain and a characteristic monsoon climate consisting of low latitude plateaus, very distinct rainy and dry seasons, and winter and spring drought. Winter and spring drought occurs frequently and is long-lasting, and has become one of the most important natural disasters affecting the social and economic development of Yunnan province, particularly in the central area. Medium and severe drought are the most common, and their impact is increasing [1]. Fire risk is increasing in Yunnan province because of climate change associated with increased drought frequency, intensity, and duration [1]. In addition, Yunnan province has high forest fire incidence, with an average of 2674 forest fires per year from 1951 to 2000 and an average annual burned forest area of 11.85 million hm^2^ [2]. Therefore, it is particularly important to study the response mechanisms of forest ecosystems to fire. This can inform guidelines for post-disaster forest reconstruction and may reduce the frequency of forest fires.

Wildfires have complex effects on vegetation and ecosystem function, ranging from minor damage to whole plant mortality and stand regeneration [3]. The impact of forest fire has been of concern to many scholars, and in-depth research has been carried out investigating its effects on the plant living environment, organ nutrition [4,5,6], physiological responses [3,7,8], and biodiversity [9,10]. The physiological mechanisms underlying plant stress responses warrant further investigation [3,11]. Fire-damaged Quercus pubescens showed increased stomatal conductivity and photosynthetic rates relative to controls [12], and fire can cause changes in fine wood structure [13]. Trees are affected by many biological and abiotic factors during growth, and their responses to environmental changes are complex. Non-structural carbohydrates (NSC), primarily soluble sugar and starch, are important for tree growth and metabolism. Their content can reflect the trees’ overall carbon balance and strategy for responding to environmental changes [14]. NSC maintain osmotic regulation, hydraulic transmission, growth, and development through their storage, transformation, and distribution among different organs of trees. They also buffer the asynchrony of carbon supply and demand between years, seasons, and organs [11]. NSC response to drought has been reported widely [15,16,17,18]. One year after forest fire disturbance, soil water content is reduced by 42%, which may induce carbon starvation and lead to tree mortality [8,13]. However, the effect of fire on NSC is less frequently studied [19,20,21,22], and the role of NSC after disturbances like fire is not well understood [11,12].

Forest fire is an important part of the biogeochemical cycle, and drives the redistribution of nutrients in forest ecosystems by directly applying carbon (C), nitrogen (N), and phosphorus (P) and changing physical, chemical, and biological soil properties [5]. Global climate change has increased forest fire frequency and intensity, which has in turn altered the nutrient balance of forest ecosystems [23]. Therefore, exploring the trophic ratios and coupling mechanisms among forest ecosystem components following forest fire disturbance has become common in forest fire ecology [24]. Understanding the mechanisms of C, N, and P cycle regulation by forest fire disturbance is also an important part of biogeochemical cycle research in the context of global change [24]. Using stoichiometry theory to study forest ecosystems after fire disturbance can not only improve our understanding of response strategy, but also contribute to the development of forest fire ecology theory [25]. Forest fire has a profound impact on nutrient balance and cycling [5], and fire factors (including intensity, frequency, years of post-fire restoration), vegetation types, and soil properties are closely related to the stoichiometric characteristics of post-fire forest ecosystems [4,26]. However, most research has focused on leaf chemometrics, and plant organs as a whole are understudied [27,28,29].

*Pinus yunnanensis* is a unique tree species in southwest China. It is widely distributed in southeastern Tibet, southwestern Sichuan, western Guizhou, western Guangxi, and most of Yunnan Province, at latitudes 23°00′–29°00′ N and longitudes 98°30′–106°00′ E. It is a pioneer tree and a major timber species for reforestation of barren mountains in southwest China, and it is one of the main components of coniferous forest vegetation in the subtropical mountainous areas of southwest China, with many ecological benefits and high economic value. *P. yunnanensis* is also a deep-rooted and tall tree species with thick bark and a perfect vascular bundle system, rendering it resistant to surface fire. If there is a fire in the distribution area, *P. yunnanensis* can be restored via “flying seeds into forests” if there are parent trees nearby. Prescribed burning is a common *P. yunnanensis* management measure and has attracted the attention of many scholars [30]. Moderate forest fire leads to burned tree layers, reduced canopy density, increased light penetration to the forest floor, increased shrub and herbaceous layer growth, and rapid forest stand recovery. Severe forest fire can destabilize forest ecosystems, affect the direction of community succession, and slow the recovery of vegetation [26]. Thermal injury suffered in a fire can initiate a complex cascade of mechanisms that affect the physiological condition of trees [8], and can interact with other disturbances such as drought. Crucially, the physiological mechanisms underlying fire-induced alterations in NSC dynamics and stoichiometric relationships of C-N-P remain underexplored, particularly with regard to their interactions with belowground carbon allocation and nutrient resorption efficiency. Addressing this critical knowledge gap is essential for advancing theoretical frameworks of post-fire biogeochemical cycling and informing ecological restoration strategies. Therefore, our objectives were to analyze whether, and in which organs, NSC and stoichiometric characteristic concentrations are changed by fires. We hypothesized that trees would not fully return to normal growth, and would have different NSC, C, N, and P concentrations nine months after wildfire. The results of the study are useful for understanding *P. yunnanensis* adaptation to fire and for scientific management after fire.

## 2. Results

### 2.1. NSC

Forest fire disturbance had significant effects on soluble sugars, starches, and NSC in each organ, and showed significant interaction effects (Table 1). Except for soluble sugars in needles, starch and NSC in stems, and sugars and NSC in branches, all indicators in the four organs were significantly affected by fire disturbance (Table 2).

**Figure 1:**
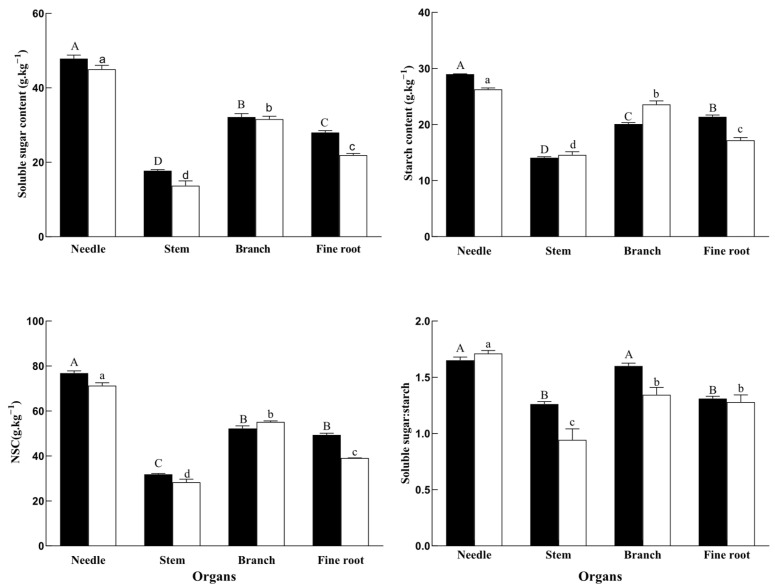
Accumulation and distribution of non-structural carbohydrates in *P. yunnanensis* among different treatments (mean ± SD). The black and white bars indicate F-trees and H-trees, respectively. Different capital letters indicate significant differences among different organs in F-trees (*p* < 0.05) and different lowercase letters indicate significant differences among different organs in H-trees (*p* < 0.05).

The contents of soluble sugars, starches, and NSC in *P. yunnanensis* organs are shown in Figure 1. After forest fire disturbance, soluble sugar content increased by 6.3%, 27.74%, and 29.39% in leaves, fine roots, and stem, respectively. Starch content increased by 10.35% and 24.51% in leaves and fine roots, respectively, while it decreased by 17.38% in branches. NSC content increased by 7.80% and 26.27% in leaves and fine roots, respectively. The ratio of soluble sugar to starch content decreased in leaves, yet increased in other organs.

The distribution of soluble sugars and NSC among the organs was consistent for F-type and H-type trees. The soluble sugar content was highest in leaves, followed by branches, fine roots, and stems. The NSC content was highest in leaves, followed by roots and branches, and was lowest in stems. The distribution of starch among the organs changed after fire exposure, and was highest in leaves and roots, followed by branches and stems (Figure 1).

### 2.2. Ecological Stoichiometry Characteristics

The two-way ANOVA (Table 3) indicated that forest fire disturbance had a highly significant effect on P, C/N, C/P, and N/P contents, but not C or N contents. Moreover, all of the data showed significant interactions. All of the data showed significant differences among organs except for C content in needles and stems, P content in stems, C/N content in branches, and C/P contents in stems (Table 4).

**Figure 2:**
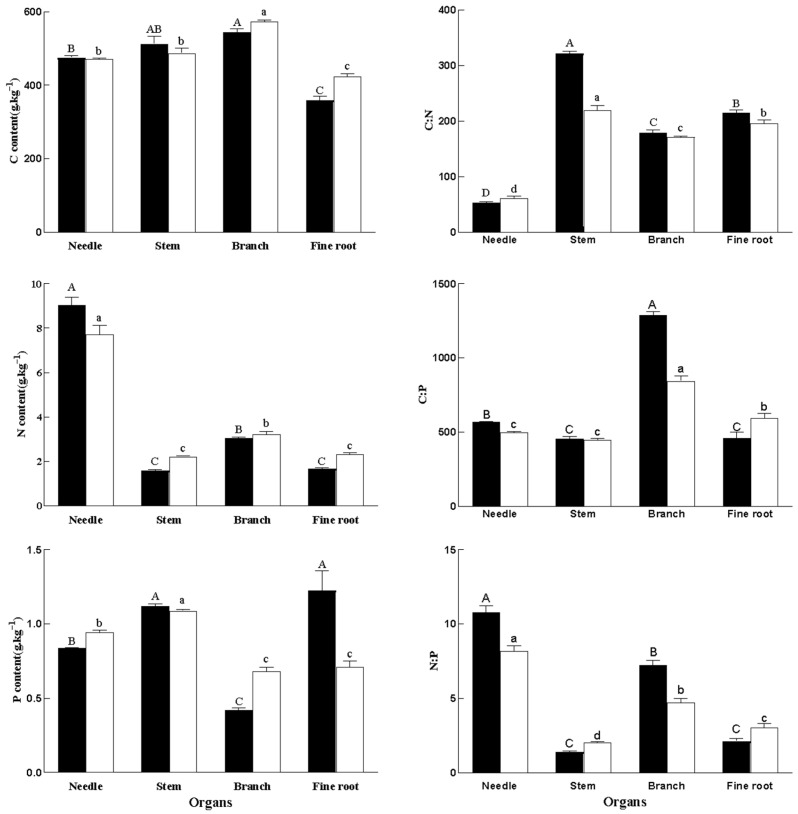
Changes in C, N, P, and their stoichiometric ratios of *P. yunnanensis* in different treatments. The black and white bars indicate F-trees and H-trees, respectively. Different capital letters indicate significant differences among different organs in F-trees (*p* < 0.05) and different lowercase letters indicate significant differences among different organs in H-trees (*p* < 0.05).

C, N, and P contents in various organs are shown in Figure 2. After fire, C content decreased by 5.08% and 14.93% in branches and fine roots, respectively. N content increased by 22.66% in leaves and decreased by 28.70% and 28.32% in stems and fine roots, respectively. P content increased by 73.23% in fine roots and decreased by 11.58% and 38.24% in leaves and branches, respectively. C/N increased by 46.99% in stems and decreased by 17.7% in leaves. C/P increased by 14.03% and 52.71% in leaves and branches, respectively, and decreased by 22.67% in fine roots. N/P increased by 38.33% and 52.71% in leaves and branches and decreased by 30.73% and 29.84% in stems and fine roots, respectively.

The distribution of C, N, C/N, and N/P in all organs was the same for F-trees and H-trees. N content was significantly higher in leaves than in all other organs, followed by branches, roots, and stems. C/N content was highest in stems, followed by roots, branches, and leaves, and was significantly lower in leaves than in all other organs. N/P content was significantly higher in leaves than in all other organs, followed by branches, roots, and stems. The distribution of P among the organs changed after forest fire disturbance, and was highest in roots, followed by stems, leaves, and branches (Figure 2).

## 3. Discussion

### 3.1. Effects of Forest Fire Disturbance on NSC Content and Distribution in P. yunnanensis Organs

The wildfire in our study occurred during the dry season, and this exacerbated the drought stress. The trees had also gone through a growing season before we took samples. In our study, after forest fire disturbance, soluble sugar content increased in leaves, fine roots, and stems. Starch content increased in leaves and fine roots. NSC content increased by 7.80% and 26.27% in leaves and fine roots, respectively (Figure 1). Our results differed from those reported after fire disturbance in different species [21,22,31] and at the organ level [11]. The significant increase in NSC content in fine roots and needles may have been because forest fires exacerbate drought, and needles, as NSC “source organs”, tend to supply NSC preferentially to the most needy organs in order to regulate their osmotic pressure and organ cavitation repair after environmental stresses [11]. Moreover, fire-induced canopy damage reduces photosynthetic capacity, triggering preferential carbon allocation to roots for storage (NSC accumulation) rather than growth. In frequently burnt mesic savanna *Acacia karroo* saplings, the concentration of starch in the roots of the burnt plants (0.08 ± 0.01 g· g^−1^) was half that of unburnt plants (0.16 ± 0.01 g· g^−1^) at the end of the first growing season after severe fire [21]. Three shrub species experiencing high mortality were depleted of starch after fire disturbance [22]. *Celastrus orbicularis* showed no significant change in root starch content after middle severity fire disturbance [31]. After high severity fire and drought, the starch content of burnt *Adenostoma fasciculatum* leaves did not change, whereas the starch content of burnt *Heteromeles arbutifolia* decreased [22]. These results showed that the starch and NSC responses to fire vary across plant species and organs [32].

NSC respond differently to drought stress and re-watering in different tree organs [33,34]. NSC content did not return to control level after extreme drought and re-watering in Chinese fir (*Cunninghamia lanceolata*) saplings [34]. Different drought intensities had different effects on *Pinus tabulaeformis* seedlings after 5 and 25 days after re-watering, and the starch and soluble sugar content of roots and leaves did not decrease under severe and moderate drought stress [30]. In this study, the starch, sugar, and NSC content of roots and needles in mature trees increased, and the NSC content of leaves and roots did not return to control level nine months after fire and drought stress (Figure 1). Our results were contrary to those shown in seedlings and saplings of *C. lanceolata* [34] and *P. tabulaeformis* [30]. This may be due to differences in the age of the trees [35]. Moreover, these studies were based on potted trees under unnatural conditions, and may therefore fail to predict responses of trees under natural conditions [11]. In brief, both severity and growth stage should be considered in assessing recovery effects after fire and drought disturbance.

The allocation of NSC in plant organs depends on the ability of each organ to utilize them, and is mainly determined by the allocation of photosynthetic products fixed by leaf uptake in different organs and transported to specific organs as structural carbon (for tissue growth) and NSC (for storage). In this study, the distribution of soluble sugars in different *P. yunnanensis* organs was consistent in F-trees and H-trees, while the distributions of starches and NSC were altered by fire (Figure 1). NSC content was highest in needles, followed by roots, branches, and stems (Figure 1). This is consistent with the distribution of NSC observed in *Larix gmelinii*, *Q. mongolica*, and *Pinus koraiensis* after fire, probably because the fire caused nutrient deficiency in the trees and changed the distribution of nutrients in the organs. This study showed that after fire disturbance *P. yunnanensis* invests non-structural carbon into the growth of leaves and fine roots. This promotes photosynthesis in the needles in order to maintain the plant’s growth and enhances fine root metabolic activity, which increases water and nutrient absorption in response to the altered external environment and nutrient deficiency.

### 3.2. Effects of Forest Fire Disturbance on Chemometric Characteristics in P. yunnanensis

The influence of forest fire on C-N-P stoichiometric characteristics of forest ecosystems is related to fire attributes (intensity, frequency, and recovery time after fire), vegetation types, and soil properties, and the fluctuation of C, N, and P in both time and space has far-reaching impact on plant nutrient absorption. Our study found that after forest fire disturbance, C content in the branches and fine roots of *P. yunnanensis* was lower, while C content in needles and stems was higher (Figure 2). This may be due to the reduction in stand canopy density and the increase in light transmittance in forests after fire, as these may enhance the C fixation capacity of stems and leaves. Moreover, it may be that the increase in C content in needles is itself a reaction to forest fire disturbance, because higher C content in plant leaves results in stronger ability to resist external environmental stress. The decrease in C content in branches and fine roots may have occurred because forest fire changed the trees’ normal physiological activities and promoted the growth of fine roots and branches. As the primary organ for absorbing water and nutrients, fine roots have high physiological activity, and their C content decreased due to the increase in lignification as the diameter of fine roots increased. This is consistent with changes in C content of *Larix gmelinii* roots after forest fire [36].

This study also demonstrated that N content in needles increased significantly after fire disturbance, while N content in all other organs decreased (Figure 2). This indicated that forest fires caused the loss of water, soil, and litter from the forest floor, and this reduced its total N content [36]. Plants invest N from other organs to protect photosynthetic organs and maintain photosynthetic production of needles in order to adapt to low nitrogen environments. In this study, P content in needles and branches decreased after fire disturbance, while P content in stems and fine roots increased after fire disturbance (Figure 2). This phenomenon may be attributed to the heightened phosphorus P demand for the growth and recovery of fine root systems and stems following fire disturbance, leading to the translocation of P from foliage to these critical regenerative organs.

In our study, C/N increased by 46.99% in the stems and decreased by 17.7% in the leaves (Figure 2). C/P increased by 14.03% and 52.71% in the leaves and branches, respectively, and decreased by 22.67% in the fine roots. N/P increased by 38.33% and 52.71% in the needles and branches and decreased by 30.73% and 29.84% in the stems and fine roots, respectively (Figure 2). These results were consistent with *Eucalyptus crebra*, in which fire significantly reduced leaf C/N. In addition, C/P and N/P of leaves after fire were significantly higher than C/N [32]. C/N of plant leaves increased significantly after fire in a plateau meadow in China, but fire did not change the soil state of N [37]. These results are inconsistent with our observations. The changes in leaf C/N and N/P observed in this study were not consistent with results reported for *P. massoniana Lamb*. and *Q. acutissima Carruth*. in a mixed forest, yet changes in leaf C/P were consistent. Forest fire increased C/N in shrub and tree leaves by 18.09% and 10.02%, respectively, one year after fire in the transition zone of north subtropical and warm temperate China. C/P of shrub leaves increased by 6.67%, while N/P decreased by 13.5%, and tree leaf N/P decreased by 15.45%. Fire did not change plant leaf N/P in temperate grassland and wasteland ecosystems, and researchers believed that this was due to the simultaneous increase in plant N and P content after fire [27,28]. Moreover, in a savanna ecosystem, fire disturbance had no significant effect on the ecological stoichiometric characteristics of plants [29]. Post-fire chemometric characteristics may differ between forest and grassland ecosystems.

The impact of forest fire on forest ecosystems is long-term, and the characteristics of ecological stoichiometry differ over time after a fire occurs. This study included samples extracted nine months (one growing season) after fire disturbance, therefore the results could reflect a short-term recovery. In the early stage after fire, nutrients surge, and this is conducive to vegetation restoration [38]. Changes in the C, N, and P content in soil affect the coupling of C, N, and P in plants. A study investigating ecological stoichiometry of the lower leaves of an Australian eucalyptus forest after a planned fire found that although the planned fire did not burn the trees, the increase in soil nutrients due to ash deposition had a long-term (>4a) impact on the ecological stoichiometry and nutrient absorption mode of the leaves [39]. In severely burned areas, forest fire has a long-term and lasting (>16a) impact on the stoichiometric characteristics of litter and soil despite that little litter remains and the combustible bed structure is thin with the restoration and renewal of vegetation [23]. It is necessary to strengthen long-term research on the entire fire recovery process.

Forest fire significantly affects the physical and chemical properties of the soil and changes nutrient partitioning in the ecosystem thereby disrupting the nutrient balance in plants. The C, N, C/N, and N/P contents of F-trees and H-trees in this study were consistent (Figure 2), which indicates that forest fire had no effect on the distribution of C and N among the organs. However, P content and C/P organ distribution were affected by fire. The P content in F-trees was highest in fine roots, followed by stems, leaves, and branches, while the P content in H-trees was highest in stems, followed by leaves, fine roots, and branches (Figure 2). This indicated that forest fire affected the fine roots of *P. yunnanensis* and more P was allocated to post-disaster repair of the fine root system, fine root morphology, fine root secretion, etc. [37].

Plant C/N and C/P can directly reflect growth rate and C fixation efficiency, and higher values indicate high nutrient use efficiency. The “growth rate hypothesis” suggests that plants with high growth rates have lower C/N and C/P [37]. Moreover, N/P determines community structure and function and can indicate nutrient limitation [37]. Plant growth was mainly limited by P when N/P of leaves was 14–16, and plant growth was limited by both N and P [40]. In this study, the mean N/P of F-tree needles were 10.9, and the mean N/P of H-tree needles was 8.16, indicating that the growth of both groups was limited by N rather than P.

C/N of F-tree leaves was lower than that of H-trees, while C/P of F-tree leaves were higher than that of H-trees. C/N of F-tree fine roots was higher than that of F-trees, yet C/P of F-tree fine roots was lower than that of H-trees. Our results indicated decreased N and increased P utilization in needles after fire. The efficiency of N utilization by fine roots increased after fire, yet the efficiency of P utilization by fine roots decreased. This is consistent with results of a chemometric analysis of *P. yunnanensis* in the same area [41].

### 3.3. Implications for Vegetation Restoration

After the understory vegetation was burned, the forest floor became exposed and loosened. Combined with the steep slope of this area, this creates high susceptibility to soil erosion during the rainy season. Additionally, the regeneration of understory vegetation is poor. Therefore, the primary objective in this area is to restore the understory vegetation. Based on practices observed in other *P. yunnanensis* forests following fires in Yunnan Province, native tree species are the preferred choice [42,43]. This study indicates that the fire has exacerbated nitrogen limitation. Therefore, shade-tolerant species such as *Alnus nepalensis* should be planted within the burned area. *Alnus nepalensis* is also a nitrogen-fixing species that grows rapidly, significantly increasing soil nitrogen content after planting. Additionally, replanting *Quercus acutissima Carruth* and *Quercus variabilis* Bl will facilitate the future establishment of mixed coniferous–broadleaf forests. The decomposition rate of coniferous and broadleaf litter under such mixed canopies is faster than in *P. yunnanensis* monoculture stands.

## 4. Materials and Methods

### 4.1. Study Site and Species

Yongren County is located in the north of Chuxiong Yi Autonomous Prefecture, Yunnan province between 101°19′–101°52′ E and 25°52′–26°32′ N. Baimahe forest is located between 101°27′–101°28′ E and 26°11′–26°25′ N. The average altitude is 1900–2775.5 m and the average slope is 20–25°. The climate is temperate and warm, with distinct dry winters and springs and wet summers and autumns. The maximum temperature is 34.7 °C, the average annual temperature is 14.8 °C, the annual rainfall at approximately 1000 mm, and the humidity is 73%. The main soil type in the area is red loam soil.

The Baimahe forest area of the state-owned forest farm in Yongren County has a total operating area of 10,300 hm^2^. It is a state-owned forest farm focusing on forest management and protection. The dominant tree species is *P. yunnanensis*, accounting for >80% of the trees in the area. *Quercus variabilis*, *Cyclobalanopsis glaucoides*, *Quercus franchetii*, and *Cyclobalanopsis delavayi* account for 10–20%. The forest is mainly a middle-aged plantation.

### 4.2. Plot Setting

In March 2019, a surface forest fire occurred on Qiaodi mountain, Yongxing Township, Yongren County. Wind speeds at the forest fire site reached force 4 to 5 on the Beaufort scale. The fire progressed rapidly as a surface fire across terrain with slopes of 50 to 60 degrees. The densely vegetated area was dominated by *P. yunnanensis* and understory shrubs, fueling intense flames. Due to strong winds and high wind speeds, the blaze proved difficult to control. The raging fire burned for nearly 10 h before being extinguished. All shrubs and herbaceous communities in the burned area were burned, tree trunks were blackened below 5 m, and needle leaves under the canopy turned yellow.

**Figure 3:**
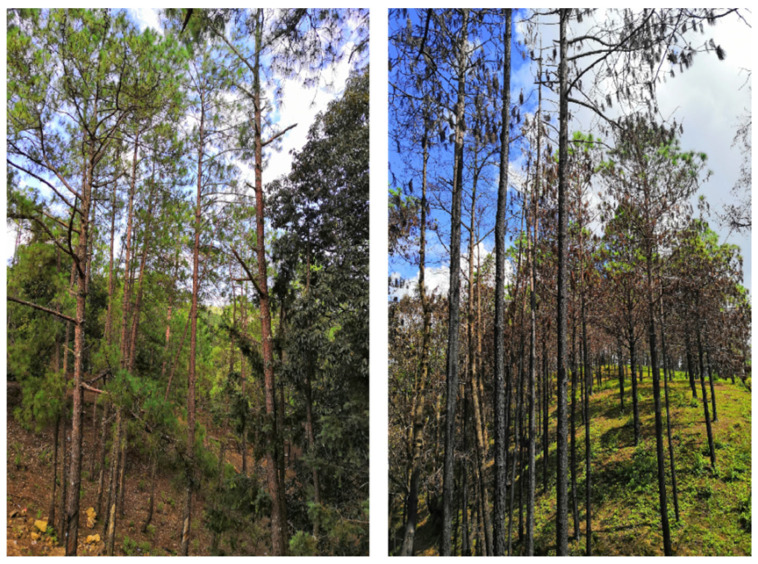
*P. yunnanensis* after forest fire disturbance (H-trees on left and F-trees on right).

Characteristics of the fire are shown in Figure 3. The fire was high-intensity (>3 m flame height) and there was no intervention afterward (Figure 3). In January 2020, three sample plots were set up in fire-burned and non-fire-burned stands, respectively (Table 5). The sample plots were 25.82 m^2^ in size, and each area was monitored and surveyed. The fire-damaged (F-trees) and undamaged (H-trees) trees were connected, and the stand characteristics were similar. The F-trees and H-trees were similar age and size.

### 4.3. Sample Collection and Measurement

Samples were collected in January 2020. In each sample plot, select stands that are representative of average growth conditions. Three standard trees were selected from each plot; a total of 18 trees were sampled from the burned and non-burned stands. The main objective is to study the effect of fire on the physiological properties of various organs, needles, stems, branches, and fine roots (diameter < 2 mm) sampled from each tree. Branches were taken from the outer, inner, and middle layers of the canopy using high branch shears, and samples from different locations in the canopy were mixed and used as individual tree needle and branch samples. A 5 mm inner diameter growth cone was used to drill cores at 1 m height in vertical direction, and the bark was removed and mixed to form trunk samples. Fine roots were sampled via digging. All samples were obtained in the field and sealed immediately after labeling.

After the samples were transported to the laboratory, they were cleaned, and put into the oven at 120 °C for 30 min. Next, they were dried at 65 °C until they reached a constant weight and were crushed and ground through a 100 mesh sieve, and put into sealed bags for measurement. Soluble sugars and starch were measured using the concentrated sulfuric acid–phenol method [35,44]. The ground sample (0.05 g) was mixed with 10 mL of distilled water and boiled in a water bath for 10 min. After centrifugation at 4000 r·min^−1^ for 10 min, the supernatant was collected. The absorbance of soluble sugars in the supernatant was then measured at 625 nm using the anthrone method with a UV-visible spectrophotometer, and their content was calculated. The resulting precipitate was retained for the determination of starch content [35,44]. Starch concentrations were determined via enzymatic hydrolysis followed by a spectrophotometric assay using glucose standard calibration curves. Organ-specific soluble sugars (ethanol-extracted) and starch (acid-hydrolyzed) were quantified separately. Total NSC were calculated as follows: NSC (g/kg DW) = [Soluble sugars] + [Starch], with triplicate measurements per organ performed using a microplate reader. Total C content was measured primarily using the K_2_Cr_2_O_7_-concentrated H_2_SO_4_ heating method, determined by spectrophotometry [45]. Total N content was measured using the Kjeldahl method; the main method involves digestion using concentrated H_2_SO_4_ (98%) and H_2_O_2_ (30%), followed by determination via the colorimetric method [46]. Total P content was determined by digestion with concentrated H_2_SO_4_ (98%) and concentrated HClO_4_ (70%), followed by molybdenum–antimony colorimetric analysis [46].

### 4.4. Statistical Analyses

Independent sample *t*-tests were used to compare the differences in the same index of the same organ in burned and unburned stands. Duncan’s multiple comparative analysis of variance (ANOVA) were used to analyze the accumulation and distribution of NSC in various organs and the influences of C, N, and P (α = 0.05). The data were tested for normality and homogeneity of variance prior to analysis of variance. All data were analyzed and figures made using SPSS 22.0 (SPSS Inc., Chicago, IL, USA) and origin 9.1 (2021 Edition, Origin Lab Company, Hampton, NH, USA). The leaves, stems, branches, and fine roots collected from each standard tree were mixed and treated as one replicate, the average value of three standard trees was treated as one quadrat replicate, and three quadrats were three replicates. The data is reported as mean ± standard deviation.

## 5. Conclusions

Forest fire disturbance significantly increased NSC content in leaves and roots of *P. yunnanensis* and altered the C, N, and P contents of each organ. Needle N/P values of both F-trees and H-trees were <14 and increased after forest fire disturbance, indicating increased N-element limitation after forest fire disturbance. Controlled application of nitrogen fertilizer can accelerate post-fire forest recovery.

*P. yunnanensis* did not return to normal physiological status nine months after the fire, indicating that more time is necessary for recovery. Forest management measures to increase soil N content may shorten post-fire recovery time. In future management of *P. yunnanensis* forests, prioritizing wildfire prevention and control is critical to mitigate fire occurrence. Our findings reveal that wildfires exert substantial impacts on these ecosystems, requiring an extended recovery period to resume normal growth trajectories.

Due to the increased frequency of fire events in different ecosystems, a better understanding of tree–fire–climate interactions and physiological responses of trees to fire disturbances is urgently required. We do not know when forest fires occur and it is difficult to simulate them experimentally; however, once they occur, we can observe the entire recovery process. This is of great value in predicting the effects of environmental changes on tree function. Future research should further investigate the post-fire recovery process. Our research relied on samples collected nine months after a fire disturbance, and longer term observation is needed. In addition, further research should include more comprehensive analyses of fire disturbance, including light, soil moisture and nutrients, ecophysiological modifications, and anatomical adaptations such as photosynthesis, carbon balance, nutrient cycling, and water-use efficiency.

## Figures and Tables

**Table 1:** Two-way ANOVA (F-value) on the effect of forest fire on the content of non-structural carbohydrates in different organs.

Factors	Soluble Sugar	Starch	NSC
Forest fire	32.119 **	6.833 *	39.251 **
Organ	469.289 **	400.407 **	792.687 **
Forest fire × Organ	3.730 *	38.455 **	17.481 **

Note: ** and * denote *p* < 0.01 and *p* < 0.05, respectively.

**Table 2:** Independent sample *t*-test analysis of the effect of forest fire on the content of non-structural carbohydrates in different organs.

Index	T Value			
	Needle	Stem	Branch	Fine Roots
Soluble sugar	2.02	3.059 *	0.458	8.814 **
Starch	10.287 **	−0.881	−5.106 **	7.209 **
NSC	3.384 *	2.430	−2.357	14.062 **

Note: ** and * denote *p* < 0.01 and *p* < 0.05, respectively.

**Table 3:** Two-factor ANOVA (F-value) of forest fire on C, N, and P contents of different organs and their stoichiometric ratios.

Factors	C	N	P	C:N	C:P	N:P
Forest fire	4.439	0.002	14,114.000 **	79.705 **	35.784 **	22.695 **
Organ	91.100 **	469.660 **	179.963 **	701.603 **	303.554 **	352.101 **
Forest fire × Organ	7.122 **	9.920 **	18.710 **	54.528 **	57.556 **	26.164 **

Note: ** denote *p* < 0.01.

**Table 4:** Independent sample *t*-test analysis of the effects of forest fire on C, N, and P contents in different organs and their stoichiometric ratios.

Index	T Value			
	Needle	Stem	Branch	Fine Roots
C	1.056	1.050	−3.320 *	−4.924 **
N	4.056 *	−11.157 **	−1.235	−8.361 **
P	−9.059 *	1.942	−7.836 **	3.788 *
C:N	−3.865 *	10.932 **	1.424	2.612
C:P	8.913 **	0.586	11.158 **	−6.405 *
N:P	5.861 **	−13.628 **	6.251 **	−5.930 **

Note: ** and * denote *p* < 0.01 and *p* < 0.05, respectively.

**Table 5:** The survey of sample site stand.

Sample Site Type	Slope Orientation	Forest Stand Depression	Average Tree Age	Average Tree Height/m	Average DBH /cm
Fire-damaged trees (F-trees)	Sunward slope	0.8	28	15.7	15.4
Undamaged trees (H-trees)	Sunward slope	0.8	28	14.2	14.9

## Data Availability

The data supporting this study’s findings are available on request from the corresponding authors.
